# Permanent health education in the context of obesity: a scoping review

**DOI:** 10.11606/s1518-8787.2023057004244

**Published:** 2023-03-15

**Authors:** Carolina Gusmão Magalhães, Ricardo Burg Ceccim, Lígia Amparo Santos da Silva, Verena Macedo Santos, Emile Miranda Pereira, Ana Artur Francisco Mussa Santos, Gesner Franscisco Xavier, Poliana Cardoso Martins, Mônica Leila Portela de Santana

**Affiliations:** I Universidade Federal do Recôncavo da Bahia Centro de Ciências da Saúde Departamento de Saúde Coletiva Santo Antônio de Jesus BA Brasil Universidade Federal do Recôncavo da Bahia. Centro de Ciências da Saúde. Departamento de Saúde Coletiva. Santo Antônio de Jesus, BA, Brasil; II Universidade Federal do Rio Grande do Sul Faculdade de Educação Programa de Pós-Graduação em Educação Porto Alegre RS Brasil Universidade Federal do Rio Grande do Sul. Faculdade de Educação. Programa de Pós-Graduação em Educação. Porto Alegre, RS, Brasil; III Universidade Federal da Bahia Escola de Nutrição Departamento de Nutrição Salvador BA Brasil Universidade Federal da Bahia. Escola de Nutrição. Departamento de Nutrição. Salvador, BA, Brasil; IV Universidade Federal da Bahia Escola de Nutrição Programa de Pós-Graduação em Alimentos, Nutrição e Saúde Salvador BA Brasil Universidade Federal da Bahia. Escola de Nutrição. Programa de Pós-Graduação em Alimentos, Nutrição e Saúde. Salvador, BA, Brasil; V Universidade Federal de Minas Gerais Faculdade de Medicina Biblioteca do Campus Saúde Belo Horizonte MG Brasil Universidade Federal de Minas Gerais. Faculdade de Medicina. Biblioteca do Campus Saúde. Belo Horizonte, MG, Brasil; VI Universidade Federal da Bahia Instituto Multidisciplinar de Saúde Departamento de Nutrição Salvador BA Brasil Universidade Federal da Bahia. Instituto Multidisciplinar de Saúde. Departamento de Nutrição. Salvador, BA, Brasil

**Keywords:** Patient Care Team, Education, Continuing, Obesity Management, Review

## Abstract

**OBJETIVE:**

To map the international literature on Permanent Health Education initiatives to care for people with obesity.

**METHODS:**

In total, six databases were searched without any language or publication period restriction according to the Joana Briggs Institute manual for evidence synthesis and the Prisma extension for scoping reviews (Prisma-ScR). Articles were independently analyzed by four reviewers and data, by two authors, which were then analyzed and discussed with our research team.

**RESULTS:**

After screening 8,780 titles/abstracts and 26 full texts, 10studies met our eligibility criteria. We extracted data on methodologies, themes, definitions of obesity, outcomes, and gaps. Most initiatives came from North American countries without free or universal health systems and lasted a short period of time (70%), had multidisciplinary teams (70%), and addressed sub-themes on obesity approaches (90%). Results included changes in participants’ understanding, attitude, and procedures (80%) and gaps which pointed to the sustainability of these changes (80%).

**CONCLUSION:**

This review shows the scarce research in the area and a general design of poorly effective initiatives, with traditional teaching methodologies based on information transmission techniques, the understanding of obesity as a disease and a public health problem, punctual actions, disciplinary fragmentation alien to the daily work centrality, and failure to recognize problems and territory as knowledge triggers and to focus on health care networks, line of care, the integrality of care, and food and body cultures.

## INTRODUCTION

In recent decades, the phenomenon of obesity has taken the center of the debate on global health concerns due to its complex and multifactorial character. In a recent report, the Lancet Commission on obesity offers the concept of a global syndemic, tracing an association between three pandemics: obesity, malnutrition, and climate change, phenomena with common systemic factors and complex relations interacting with each other to contribute to anew narrative needed to accelerate the social movement of change^1^.

A global panorama of this magnitude invites policymakers, researchers, managers, and healthcare providers to think about new forms of care which are articulated in a network, structured in longitudinal lines, and based on the integrality of care and body and food cultures^2 , 3^. Guidelines to organize obesity care lines point to Permanent Health Education (PHE) actions, programs, and primary care policies as a powerful political-pedagogical strategy to change health care providers’ understanding, formulation, and thinking, turning territorial realities into beacons for critical, systemic, and transformative actions^2^.

Thus, this review aims to systematically map and summarize the evidence found in this area via the following research question (RQ): what has the international literature produced about permanent education initiatives so health care providers can care for people with obesity? It also explores the following sub-issues:(RQ1) What themes and methodologies do these initiatives use?; (RQ2) How do these studies conceptualize obesity?; (RQ3) What are the results of these initiatives?; and (RQ4) What gaps do these studies show?

## METHODS

### Protocol and Registration

The protocol of this review was elaborated according to Prisma-P^6^ and the Joana Briggs Institute (JBI)^7^ recommendations, which were reviewed by our research team. Its final draft was registered on Open Science Framework on February 24, 2021(https://osf.io/g5jkh/).

### Eligibility Criteria

Studies (which could have included articles from peer-reviewed journals, dissertations, theses, and reviews) dealing with permanent education initiatives for the primary care of people with obesity (overweight and obese) were assessed by this review. Language or year of publication restrictions were ignored to expand the number of retrieved studies. Quantitative, qualitative, and mixed studies were included to map different methodological approaches.

Studies which met the following criteria were excluded: participants who were not primarily healthcare providers; initiatives ignoring PHE, i.e., if they had no intention of educating professionals to work in health care networks; those outside primary care; and educational initiatives which dealt with issues other than obesity. *Invitro* studies with animals, texts, opinion papers, letters, conference summaries, and editorials were excluded because they failed to meet the objectives of this review.

### Information Sources

**Figure 1:**
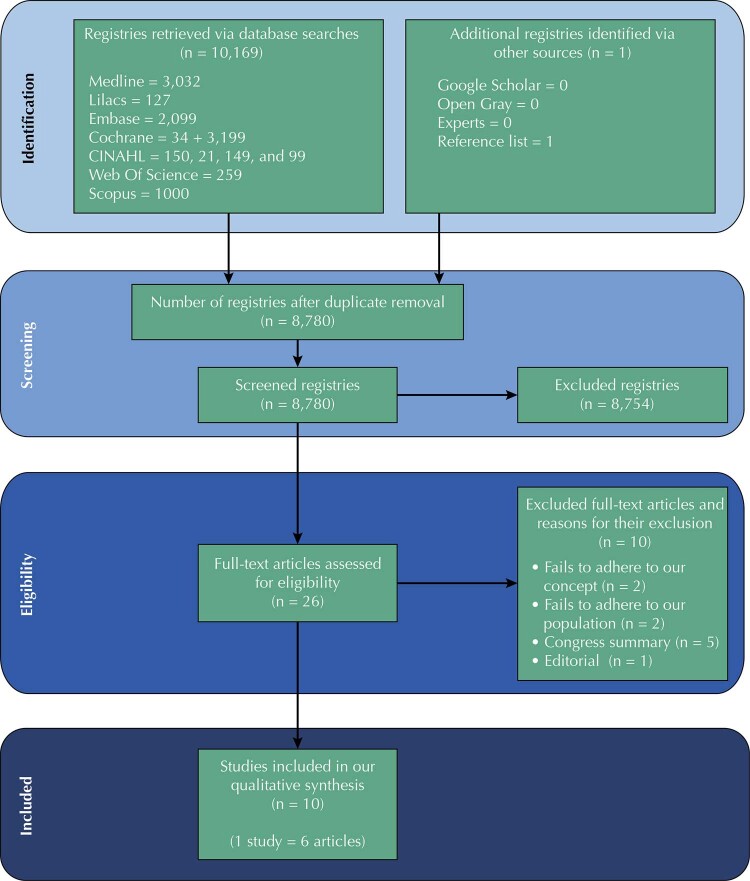
Prisma diagram flow for this scoping review.

At first, a limited search was conducted on two databases, Medline (PubMed) and CINAHL, followed by an analysis of the words contained in the title and abstract of the retrieved articles and of the terms in the indices and keywords used to describe the articles. To find as much relevant evidence as possible, our search for studies was comprehensive and systematic. Medline via PubMed, Embase, CINAHL, Scopus, Web of Science, and Lilacs were the databases selected for research. Nevertheless, Google Scholar and Open Gray were also searched for grey literature. Our search strategy was developed with descriptors which were synonymous to those in MeSH (PubMed), DeCS (Lilacs), and Emtree (Embase). Our search strategy was applied to these data bases by a reviewer and results, exported to Rayyan for duplicate removal and initial sorting. Reference lists with the included studies were evaluated. A wide consultation with specialists in the area was carried out to recover important studies which our searches failed to retrieve. Subsequently, the selected publications were exported to EndNote web. Our research team was assisted in this process by a librarian specialized in health (GFXJ). The search strategies developed for each database are included in an additional file ( Figure 1 ).

### Sources of Evidence Selection

The entire selection was accompanied by two pilot tests. On the first stage, 25 titles/abstracts from sources of evidence were read and, on the second, five others, in full. Random sample were included in both sources. These studies were screened by our entire team according to our eligibility criteria, showing an agreement above 75%, as per the JBI Manual for Evidence Synthesis.^7^Selections based on titles/abstracts and full texts were made in Rayyan by four independent reviewers (CGM, VMS, EMP, and AAFMS) and disagreements were resolved in pairs or by a third reviewer’s intervention. In the absence of additional information or data, the authors of the chosen studies were contacted.

### Data Items and Data Charting Process

Data were summarized by the following characteristics: articles (country of origin, health system, funding, year of publication and authors); participants (demographic and sample size); educational initiatives (methodology, theme addressed, results achieved and gaps); and research (context, outcome and main results). The JBI Sumari software was used to map our findings.

The entire data extraction process was carried out by two independent reviewers (CGM and VMS), who discussed results and procedurally and simultaneously updated our data graph. To increase consistency among reviewers, a pilot source extraction test was performed with a random sample of four publications, about which reviewers showed an agreement above 75% on the mapped data. Any divergence was resolved by consensus or by the intervention of a third reviewer.

### Result Synthesis

Results were synthesized by frequency and thematic analysis. Textual data were thematically analyzed by a reviewer and verified by a second one (CGM and MLPS) via ATLAS.ti. A tabular diagram, word cloud (Wordcloud.com), data graph, and narrative summary were elaborated by the authors according to Prisma ScR^8^ recommendations, both in line and describing how results related to the questions and objectives of this review.

## RESULTS (N = 10)

### Sources of Evidence Selection

We retrieved 10,169 publications, one of which^9^ by a survey in our reference lists. We removed 1390 duplicates and evaluated 8,780 titles and abstracts and 26 full-text articles for eligibility. We neither found documents in the grey literature nor by consulting specialists. Subsequently, 10 studies, reported in 15 publications, met our eligibility criteria^9^. Figure 1 shows the reasons for excluding full articles.

### Sources of Evidence Characteristics

All studies were published after 2004 and originated in the United States of America (60%)^10 , 12 , 13 , 16^, Canada (20%)^15 , 19^, Israel (10%)^11^, and Iran (10%)^14^ (Appendix S2). In total, eight publications were linked to three mixed studies^11 , 13 , 15^, seven were quantitative studies^10 , 12 , 14 , 16^, of which one was a dissertation^18^. Most were original studies (100%)^10^ conducted in countries without universal health systems (60%)^10 , 12 , 13 , 16^ which received public funding (30%)^10 , 12 , 16^, appeared in journals whose central scope addressed training healthcare providers (30%)^11 , 14 , 19^, had quasi-experimental methods (60%)^10^aimed at primary care (80%)^11^, and contained multidisciplinary groups (70%)^10 , 11 , 13 , 15 , 16 , 18 , 19^. Table 2 described the included studies.

**Table 2:** Thematic analysis results of the scoping review research questions

RQ1A. Themes of permanent education initiatives			

Category		Conts (%)	Authors (year)
Approaches to obesity	Environmental approach	2 (20%)	Stark et al. (2011)^12^; Mainor et al. (2014)^16^
	Biomedical approach	5 (50%)	Katz et al. (2005)^11^; Sarayani et al. (2012)^14^; Campbell-Scherer et al. (2014)^15^; Vasudevan et al. (2016)^17^; Joshua (2017)^18^
	Cultural/identity approach	2 (20%)	Campbell-Scherer et al. (2014)^15^; Joshua (2017)^18^
	Psychological/behavioral approach	4 (40%)	Katz et al. (2005)^11^; Stark et al. (2011)^12^; Campbell-Scherer et al. (2014)^15^; Sanchez-Ramirez et al. (2018)^19^
	Ecological approach	1 (10%)	Stark et al. (2011)^12^
Professional qualification strategies	Curriculum and competency-based training	2 (20%)	Mainor et al. (2014)^16^; Joshua (2017)^18^
	Strategic development of an intervention plan	2 (20%)	Stark et al. (2011)^12^; McPherson et al. (2012)^13^
Obesity management	Barriers and solutions in tackling obesity	1 (10%)	Whitaker et al. (2004)^10^
	Care for caregivers	1 (10%)	Campbell-Scherer et al. (2014)^15^
	Monitoring and evaluation of results in obesity management	1 (10%)	Stark et al. (2011)^12^
	Administrative and political factors whichinfluence obesity interventions	1 (10%)	Stark et al. (2011)^12^
	Prevention of obesity acrosspolicy	1 (10%)	McPherson et al. (2012)^13^
	Advocacy resources on obesity care	1 (10%)	McPherson et al. (2012)^13^
Sociohistorical overview of obesity	World obesity crisis	1 (10%)	Sanchez-Ramirez et al. (2018)^19^
	The historical trajectory of care practices for people with obesity	1 (10%)	Sanchez-Ramirez et al. (2018)^19^
Professional work style	Interdisciplinary work in obesity care^19^	1 (10%)	Sanchez-Ramirez et al. (2018)^19^

**RQ1B. Methodologies of permanent education initiatives**			

**Category**		**Conts (%)**	**Authors (year)**

Teaching strategy	Assigned readings	1 (10%)	Stark et al. (2011)^12^
	Round tables	1 (10%)	Mainor et al. (2014)^16^
	Audio-visual resources (film, documentary, webcast)	2 (20%)	Whitaker et al. (2004)^10^; Stark et al. (2011)^12^
	Sessions of skills development, training application	2 (20%)	Mainor et al. (2014)^16^; Vasudevan et al. (2016)^17^
	Support from teachers, feedback from experts, or tutoring with experienced providers	2 (20%)	McPherson et al. (2012)^13^; Sarayani et al. (2012)^14^
	Building interventions plans	2 (20%)	Stark et al. (2011)^12^; McPherson et al. (2012)^13^
	Assessment session - immediate post-intervention (after 3 and 6 months)	2 (20%)	Campbell-Scherer et al. (2014)^15^; Vasudevan et al. (2016)^17^
	Critical reviews of medical records or provider x patient/family interactions, simulated patients	2 (20%)	Sarayani et al. (2012)^14^; Vasudevan et al. (2016)
	Workshops	4 (40%)	Katz et al. (2005)^11^; Stark et al. (2011)^12^; Campbell-Scherer et al. (2014)^15^; Sanchez-Ramirez et al. (2018)^19^
	Delivery of teaching material	3 (30%)	Sarayani et al. (2012)^14^; Campbell-Scherer et al. (2014)^15^; Vasudevan et al. (2016)^17^
	Case study/field history	5 (50%)	Whitaker et al. (2004)^10^; Katz et al. (2005)^11^; Sarayani et al. (2012)^14^; Mainor et al. (2014)^16^; Sanchez-Ramirez et al. (2018)^19^
	Discussion panel (clinical reports and case presentation), discussion forums, and plenaries	6 (60%)	Whitaker et al. (2004)^10^; Katz et al. (2005)^11^; Stark et al. (2011)^12^; Sarayani et al. (2012)^14^; Mainor et al. (2014)^16^; Sanchez-Ramirez et al. (2018)^19^
	Oral presentation (lectures and conferences)	6 (60%)	Katz et al. (2005)^11^; Stark et al. (2011)^12^; Sarayani et al. (2012)^14^; Campbell-Scherer et al. (2014)^15^; Mainor et al. (2014)^16^; Sanchez-Ramirez et al. (2018)^19^
Length of permanent health education	Days (1 to 5)	7 (70%)	Whitaker et al. (2004)^10^; McPherson et al. (2012)^13^; Sarayani et al. (2012)^14^; Mainor et al. (2014)^16^; Vasudevan et al. (2016)^17^; Joshua (2017)^18^; Sanchez-Ramirez et al. (2018)^19^
	Weeks (06)	1 (10%)	Stark et al. (2011)^12^
	Semester	2 (20%)	Katz et al. (2005)^11^; Campbell-Scherer et al. (2014)^15^
Types of participation	Individual	6 (60%)	Katz et al. (2005)^11^; Stark et al. (2011)^12^; McPherson et al. (2012)^13^; Vasudevan et al. (2016)^17^; Joshua (2017)^18^; Sanchez-Ramirez et al. (2018)^19^
	Group	2 (20%)	Whitaker et al. (2004)^10^; Campbell-Scherer et al. (2014)^15^
	Mixed	2 (20%)	Sarayani et al. (2012)^14^; Mainor et al. (2014)^16^
Frequency of meetings	Single	5 (50%)	Whitaker et al. (2004)^10^; Sarayani et al. (2012)^14^; Vasudevan et al. (2016)^17^; Joshua (2017)^18^; Sanchez-Ramirez et al. (2018)^19^
	Daily	2 (20%)	McPherson et al. (2012)^13^; Mainor et al. (2014)^16^
	Weekly	1 (10%)	Stark et al. (2011)^12^
	Biweekly	1 (10%)	Campbell-Scherer et al. (2014)^15^
	Monthly	1 (10%)	Katz et al. (2005)^11^
Teaching modality	In-person course	8 (80%)	Whitaker et al. (2004)^10^; Katz et al. (2005)^11^; Sarayani et al. (2012)^14^; Campbell-Scherer et al. (2014)^15^; Mainor et al. (2014)^16^; Vasudevan et al. (2016)^17^; Joshua (2017)^18^; Sanchez-Ramirez et al. (2018)^19^
	Semi-in-person course	1 (10%)	McPherson et al. (2012)^13^
	Distance education	1 (10%)	Stark et al. (2011)^12^
Use of data from students’ reality		2 (20%)	Stark et al. (2011)^12^; Vasudevan et al. (2016)^17^
Use of professional development programs		2 (20%)	Stark et al. (2011)^12^; Campbell-Scherer et al. (2014)^15^
Qualitative assessment of the current educational needs of the involved healthcareproviders (real-life problems vs. previous knowledge and experience)		2 (20%)	Sarayani et al. (2012)^14^; Campbell-Scherer et al. (2014)^15^

**RQ3. Results of permanent education initiatives**			

**Category**		**Conts (%)**	**Authors (year)**

Participants	Conceptual, procedural, and/or attitudinal changes on obesity care	10 (100%)	Whitaker et al. (2004)^10^; Katz et al. (2005)^11^; Stark et al. (2011)^12^; McPherson et al. (2012)^13^; Sarayani et al. (2012)^14^; Campbell-Scherer et al. (2014)^15^; Mainor et al. (2014)^16^; Vasudevan et al. (2016)^17^; Joshua (2017)^18^; Sanchez-Ramirez et al. (2018)^19^
Initiative	Limits and potential of teaching strategies	3 (30%)	Katz et al. (2005)^11^; Sarayani et al. (2012)^14^; Vasudevan et al. (2016)^17^
	High level of student satisfaction with the overall quality of the course	2 (20%)	Mainor et al. (2014)^16^; Joshua (2017)^18^
Products	Production of specific-action plans for students’ reality	2 (20%)	Stark et al. (2011)^12^; McPherson et al. (2012)^13^

**RQ4. Gaps in permanent education initiatives**			

**Category**		**Conts (%)**	**Authors (year)**

Conceptual, attitudinal, and procedural change of course participants	Sustainability of conceptual, attitudinal, and procedural change of course participants	5 (50%)	Whitaker et al. (2004)^10^; Sarayani et al. (2012)^14^; Mainor et al. (2014)^16^; Vasudevan et al. (2016)^17^; Sanchez-Ramirez et al. (2018)^19^
	Ability to identify which element (strategy or content) induces course changes	1 (10%)	Stark et al. (2011)^12^
	Study the effect of the courseon the self-efficacy of healthcareproviders for continuing education	1 (10%)	Katz et al. (2005)^11^
	Analysis of other factors whichinfluence learning	2 (20%)	Whitaker et al. (2004)^10^; Campbell-Scherer et al. (2014)^15^
Teaching strategies	Application of more powerful teaching strategies	1 (10%)	Whitaker et al. (2004)^10^
	Cost-benefit assessment of teaching strategies	2 (20%)	Sarayani et al. (2012)^14^; Mainor et al. (2014)^16^
	Compare various competency models for obesity management	1 (10%)	Joshua (2017)^18^
Course duration	Short-term for evaluating on patients and/or public policies	1 (10%)	McPherson et al. (2012)^13^

### RQ1A. Permanent Health Education Initiatives themes

Most initiatives focused on self-reported approaches to obesity (80%)^11 , 12 , 14^, including biomedical, psychological/behavioral, ecological, cultural, and environmental ones; and professional qualification strategies (40%)^12 , 13 , 16 , 18^. We must point out that twostudies^14 , 15^ qualitatively assessed the involved healthcare providers’ current educational needs to build content panels for these initiatives, a participation strategy evinced in the PHE methodological principle. Table 3 shows a table with content frequency and thematic analysis.

**Table 3:** Thematic analysis results of the research questions of this scoping review.

Line No.	Database search strategy Literature Search performed: October 21, 2020	Number of results
	1. Medline (PubMed)	
#1	“Education, Continuing”[Mesh] OR (Continuing Education)	78,336
#2	“Inservice Training”[Mesh] OR (Inservice Training) OR (On-the-Job Training) OR (On the Job Training) OR (Training, On-the-Job) OR (Training, Inservice) OR (Orientation Programs, Employee) OR (Employee Orientation Program) OR (Orientation Program, Employee) OR (Program, Employee Orientation) OR (Programs, Employee Orientation) OR (Employee Orientation Programs) OR (Health Human Resource Training)	69,070
#3	“Education”[Mesh:NoExp] OR (Education)	1,792,949
#4	#1 OR #2 OR #3	1,800,195
#5	“Health Personnel”[Mesh] OR (Personnel, Health) OR (Health Care Providers) OR (Health Care Provider) OR (Provider, Health Care) OR (Providers, Health Care) OR (Healthcare Providers) OR (Healthcare Provider) OR (Provider, Healthcare) OR (Providers, Healthcare) OR (Healthcare Workers) OR (Healthcare Worker)	765,649
#6	“Obesity”[Mesh] OR (Obesity)	372,187
#7	“Overweight”[Mesh] OR (Overweight)	250,182
#8	#6 OR #7	387,011
#9	$4 AND #5 AND #8	3,032
	2. BVS	
#1	(“Educação Continuada” OR “Education, Continuing” OR “Educación Continua” OR “Educação Contínua” OR “Educação Permanente” OR “Formação Continuada” OR “Capacitação em Serviço” OR “Inservice Training” OR “CapacitaciónenServicio” OR “Formationen interne” OR “Programas de Orientação ao Empregado” OR “Treinamento em Serviço” OR”Capacitação de Recursos Humanos em Saúde” OR “Health HumanResourceTraining” OR “Capacitación de Recursos Humanos enSalud” OR “FormationdesRessourcesenSantéHumaine” OR “Capacitação de Recursos Humanos Especializados” OR “Formação Profissional em Saúde” OR educação OR education OR educación)	-
#2	(“Pessoal de Saúde” OR “Prestadores de Cuidados de Saúde” OR “Profissionais da Saúde” OR “Profissionais de Saúde” OR “Profissional da Saúde” OR “Profissional de Saúde” OR “Trabalhador da Saúde” OR “Trabalhador de Saúde” OR “Trabalhadores da Saúde” OR “Trabalhadores de Saúde” OR “Health Personnel” OR “Health CareProviders” OR “HealthcareProviders” OR “HealthcareWorkers” OR “Personal de Salud” OR “Proveedores de Atención de Salud” OR “Trabajadores de laSalud” OR mh:m01.526.485*)	-
#3	(obesidade OR obesity OR obesidad OR “Tratamiento de la Obesidad” OR sobrepeso OR overweight OR sobrepeso)	-
#4	#1 AND #2 AND #3 (“Educação Continuada” OR “Education, Continuing” OR “Educación Continua” OR “Educação Contínua” OR “Educação Permanente” OR “Formação Continuada” OR “Capacitação em Serviço” OR “Inservice Training” OR “CapacitaciónenServicio” OR “Formationen interne” OR “Programas de Orientação ao Empregado” OR “Treinamento em Serviço” OR “Capacitação de Recursos Humanos em Saúde” OR “Health HumanResource Training” OR “Capacitación de Recursos Humanos enSalud” OR “FormationdesRessourcesenSantéHumaine” OR “Capacitação de Recursos Humanos Especializados” OR “Formação Profissional em Saúde” OR educação OR education OR educación) AND (“Pessoal de Saúde” OR “Prestadores de Cuidados de Saúde” OR “Profissionais da Saúde” OR “Profissionais de Saúde” OR “Profissional da Saúde” OR “Profissional de Saúde” OR “Trabalhador da Saúde” OR “Trabalhador de Saúde” OR “Trabalhadores da Saúde” OR “Trabalhadores de Saúde” OR “Health Personnel” OR “Health CareProviders” OR “HealthcareProviders” OR “HealthcareWorkers” OR “Personal de Salud” OR “Proveedores de Atención de Salud” OR “Trabajadores de laSalud” OR mh:m01.526.485*) AND (obesidade OR obesity OR obesidad OR “Tratamiento de laObesidad” OR sobrepeso OR overweight OR sobrepeso) AND ( db:(“LILACS” OR “IBECS” OR “BDENF” OR “INDEXPSI” OR “PERNAL” OR “BBO” OR “CUMED” OR “MedCarib” OR “PREPRINT-MEDRXIV” OR “BINACIS” OR “PREPRINT-SCIELO” OR “SES-SP” OR “colecionaSUS”))	127
	3. Embase (Emtree Descriptors)	
#1	‘continuing education’/exp OR ‘continuing education’	50,656
#2	‘in service training’/exp OR ‘in service training’	17,282
#3	education:ti,ab,kw	644,878
#4	#1 OR #2 OR #3	2,242,533
#5	‘health care personnel’/exp OR ‘health care personnel’	1,615,480
#6	‘obesity’/exp OR obesity	631,624
#9	$4 AND #5 AND #6	2,099
	4. Cochrane	
#1	MeSH descriptor: [Education, Continuing] explode all trees	
#2	(“Continuing Education”):ti,ab,kw	
#3	MeSH descriptor: [Inservice Training] explode all trees	
#4	(“Inservice Training”):ti,ab,kw	
#5	MeSH descriptor: [Education] this term only	
#6	(“education”):ti,ab,kw	
#9	MeSH descriptor: [Health Personnel] explode all trees	
	(“Health Personnel”):ti,ab,kw	
	MeSH descriptor: [Obesity] explode all trees	34 Reviews and 3,199 Trials
	5. CINAHL/Web of Science (WOS)/Scopus	
	(“Education, Continuing” OR “Continuing Education” OR “Inservice Training” OR “Health Human Resource Training” OR education) AND (“Health Personnel” OR “Health Care Providers” OR “Healthcare Providers” OR “Healthcare Workers”) AND (obesity OR overweight)	CINAHL: 470
		WOS: 259
		Scopus: 1,000

### RQ1B. Methodology of Permanent Health Education Initiatives

Most studies showed the following methodological profile for their initiatives: oral exposure (60%)^11 , 12 , 14^ and/or discussion (60%)^10^ as teaching strategies; which lasted one day (70%)^10 , 13 , 14 , 16^and included individual (80%)^11^ in-person participation (80%)^10 , 11 , 14^ in a single meeting (50%)^10 , 14 , 17^. Some initiatives used data from participants’ reality, such as the prevalence of local obesity^12^ and evaluations of medical records of patients with obesity^17^. Table 3 shows these data.

### RQ2. Concept of Obesity

**Figure 2:**
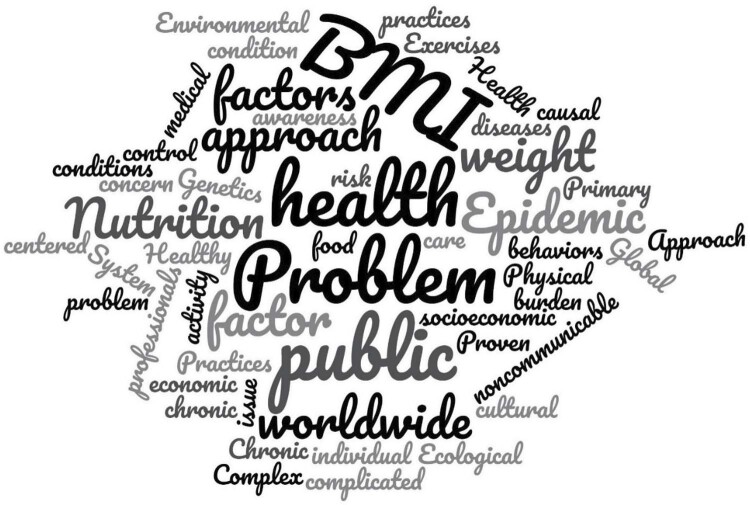
Obesity concept word cloud.

Most studies considered obesity as a problem (60%)^10 , 11 , 14 , 15 , 18 , 19^ linked to world public health (30%)^11 , 14 , 15^ and complex systems (10%)^19^ which is common in primary care (10%)^15^. The other studies brought concepts such as world epidemic (20%)^15 , 18^; prevalent chronic conditions (10%)^15^; and risk factors for non-communicable chronic diseases (20%)^17 , 19^. Studies focused on explanatory models of obesity: (i) body mass indices (BMI) (40%)^14 , 15 , 17 , 18^; (ii) the environment (20%)^12^; (iii) culture (20%)^15 , 18^; (iv) ecology (10%)^12^; and (v) the perspective of a complex system, in which individual behaviors related to nutrition and physical activity interact with genetics, socioeconomic factors, among other factors (10%)^19^. Figure 2 shows a conceptual word cloud arrangement of these explanatory models.

### RQ3. Results of Permanent Health Education Initiatives

All initiatives showed results on participants’ conceptual, procedural, and attitudinal changes (100%)^10^, evaluating them via pre-research (100%), immediate post-intervention (80%), and afterone^12 , 15^, three^17^, six^15 , 16 , 19^, and 12 months^15^. Table 3 offers a more detailed description.

### RQ4. Gaps in Permanent Health Education Initiatives

Most initiatives showed gaps related to sustaining the conceptual, attitudinal, and procedural changes promoted by their courses (50%)^10 , 14 , 16 , 17 , 19^ and teaching strategies (40%)^10 , 14 , 16 , 18^. Table 3 offers a more detailed description.

## DISCUSSION

Regarding their general scope, results (n = 10) show a scarcity of research in the area and, consequently, novelty and importance for policymakers^24^. The included studies showed initiatives to educate healthcare providers with methodological profiles focused on specific actions, technical and instrumental biases, and subthemes which addressed obesity (especially from a biomedical perspective— among others which often failed to dialogue with the problems of healthcare providers’ everyday life). Our sample addressed obesity as a public health problem, evincing a more quantitative bias between disease and risk factor. Initiative results showed changes in professionals’ concept and attitude toward obesity but only slightly altered the procedural field of caring for these patients, showing, in their gaps, the concern that future longitudinal research can investigate changes in long-term care practices.

Although widely used in its health services, we retrieved no study conducted in Latin America, in which PHE emerged in the 1980s as a methodological strategy at the initiative of the Pan American and World Health Organizations, structured in 2004 as a public policy for health training linked to national health services and disseminated throughout the Brazilian territory^25^. Thus, it is necessary to consider the importance of encouraging the scientific dissemination of experiences in the everyday life of health services as evidence which can structure major scientific leaps in the area.

The fact that most studies come from the USA^11^ endorses its pioneering and advancing spirit in scientific research on obesity handling and management. It historically adopted a mixed health system model, lacking universal health coverage and whose private sector lies higher than the public one. Moreover, it has institutionalized the issue of obesity – a process which corroborated, according to Poullain^26^, to place it as a world public health issue.

Such conditions signal a shift from the epistemological status of obesity to a more quantitative definition which suffers great interference from economic interests (private health insurance and food companies)^27 , 28^ which outline the scope of such investigations, both assuming the hegemony of biomedical biases and the “warlike” logic of dealing with obesity, which, according to Foucault^29^, controls body mass structure biopower techniques centered on the individualized body and considers it relevant to understand the tensions, logics, and interests around obesity to think of care strategies more focused on integrality, respect for individuality, and a critical position against the stigma it carries.

This review assumed a conceptual-methodological difference between continuing education (CE) and PHE. The first, the traditional resource in health, centers around updating knowledge, usually with a disciplinary focus based on transmission techniques, thus constituting a discontinuous training strategy with time breaks^25 , 30^. PHE, on the other hand, proposes changing the conception and practices of health training and incorporating teaching and learning into the daily life of services, with practice as a source of knowledge and problems that increase teams’ critical thinking based on network, comprehensive care, and multiprofessional, interdisciplinary, and intersectoral approaches^2 , 5 , 25 , 30^.In our analysis of the context of these actions, the included studies show initiatives which mainly relate to CE — punctual actions^10 , 13 , 14 , 16 , 17 , 18 , 19^, disciplinary fragmentation^11 , 16 , 17^,andtraditional teaching methodologies based on transmission techniques^10 , 11 , 13 , 16 , 18 , 19^, even if some had moved toward PHE. This methodological design exposes a still present weakness in health education between what is desired and what is effectively done. It aims, for example, to offer something new using the same and exhausted strategies of knowledge mediation.

PHE presupposes the development of educational practices focusing on the resolution of concrete problems via team discussions based on the perspective of transforming work processes^2 , 5 , 25 , 30^. Thus, four studies claimed, as their methodological strategies, to survey both participants’ problems as guidelines to build themes/contents for their PHEinitiatives^14 , 15^ and data on the health service reality^12 , 17^ and on advocacy resources in the care forobesity^13^, a strategy based on what Ceccim^2^ calls a four-way approach to training: teaching - care- management - social control, in which “each face involves a pedagogical call, an image of the future, an apolitical struggle, and a web of connections” (p.47). These strategies project participants’ critical approach to their realities, promoting the recognition of possibilities for emancipatory action in the face of the actual situations experienced in health services.

The contents of the initiatives dealt with approaches toobesity^11 , 12 , 14^, most only discussed its biomedical perspective^11 , 14 , 16 , 19^; professional qualification strategies^12 , 13 , 16 , 18^; relations with PHE, including the strategic development of intervention plans^12 , 13^; and obesity handling and management^10 , 12 , 13 , 15^. In general, initiatives mainly discussed continuous self-improvement in the search for professional and personal competence and only slightly consider the situations problematizing work to transform reality^33^. The addressed sub-themes need to dialogue more with participants’ yearnings for change since they show a greater number of possible points of analysis when facing the same situation —obesity —, subsidizing the critical analysis of their realities to promote more critical, creative, and autonomous decision-making.

Educational actions in health human resources traditionally have methodological designs consisting of short actions^10 , 13 , 14 , 16 , 17 , 18 , 19^ which favor individual participations^11 , 12 , 13 , 17 , 18 , 19^ and teaching strategies mainly based on oral exposure^11 , 12 , 14 , 15 , 16 , 19^; methodological conditions which, allied to institutional, political, ideological, and cultural ones, anticipate and determine the space within which training can operate its limits and possibilities: a simplified and instrumental vision of education; its low discrimination of problems to be overcome; and immediacy in projects with predetermined beginnings and ends^25 , 34^; results pointing to methodological designs far removed from what the National PHE Policy proposes in Brazil^25 , 32^.

Studies conceptualized obesity as a problem^10 , 11 , 14 , 15 , 18 , 19^ in global public health^11 , 14 , 15^ which has a complex system^19^ and is common in primary care^15^. Recognition as a public health issue, according to Poulain^26^, occurs after two conditions are met: theme institutionalization and the change in the epistemological status of obesity to a more quantitative definition — either as a risk factor^17 , 19^ or a disease, consolidated by the use of BMI as an evaluation method^14 , 15 , 17 , 18^ — in a process which medicalized obesity^26^, although limited, according to Gard & Wright^35^.

Since the WHO^36^ used the term global “epidemic” (a concept which two studies^15 , 18^ mention), obesity has gained prominence in the media and political debate, forming a true “warlike” scenario with considerable symbolic consequences and involving different agents which are motivated by different interests, offering questionable services, products, and information in the fight against the “enemy”^1 , 26^. Although this narrative represents the current hegemonic discourse, according to the Lancet^1^report and authors from the Social and Human Sciences^26 , 35 , 37^, it offers a limited understanding in the face of the phenomenon of obesity because it ignores its complexity, a condition only one study^19^ signaled and valued by the systemic approach of PHE.

Regarding the results of these initiatives, all studies point to changes in participants. However, the most expressive are, according to the typology in Zabala^41^, in their conceptual^10^ and attitudinal content^12 , 15 , 16 , 18 , 19^, with lower expression in the procedural one^13^, a great objective regarding primary care work collectives^31^. Moreover, some results describe the limits and potentialities of teaching strategies^10 , 14 , 17 , 18^, in which we find the success of those involving discussion and group work when we compared them to transmission strategies^10 , 14 , 17^. Thus, longitudinal studies may be a good strategy to promote more lasting changes also in the field of procedural contents installed in health services.

Also regarding results, two studies^17 , 18^ highlighted that the use of strategies to understand cultural contexts (by race) within services provided significant conceptual and procedural changes for some participants, endorsing a premise of reorganizing their work process via the concept of territorialization^42^ as an instrument to diagnose and analyze health situations in local planning. Finally, regarding the products of these actions, two studies^12 , 13^ elaborated an individual intervention plan, a strategy qualifying social actors^33^ for strategic thinking/reasoning and the ability to contextualize projects for relevant healthcare problems^43^. The latter results are the most significant and desired for health services for their transformative power and association with the real needs of the monitored population.

Regarding gaps, six studies^10 , 13 , 14 , 16 , 17 , 19^ recorded the importance of further research designing longer and more targeted initiatives to assess the sustainability of conceptual, attitudinal, and procedural changes, considering that, even if these initiatives achieve individual learning, they fail to always translate themselves into organizational learning, i.e., the reorganization of collective work processes^25 , 33^, a premise of the Brazilian National PHE Policy^25 , 32^. Some studies^10 , 14 , 16 , 18^ indicate the possibility of surveying more powerful and cost-effective teaching strategies given the exhaustion of so many traditional and discontinued strategies aimed at transmission. In this sense, the PHE launches itself as a potential political-pedagogical practice that,

(…) at the same time as it disputes for the daily updating of practices according to the most recent theoretical, methodological, scientific, and technological contributions available, it is part of a necessary construction of relationships and processes which stem from within teams in a joint action – including their agents, organizational practices – involving the institution and/or the health sector and interinstitutional and/or intersectoral practices – linked to the policies in which the health acts are included^33^ (p.161).

Interpreting international production shows that obesity is a public health problem, and that health education rarely questions food versus body. Bio pedagogical studies on the stigma of obesity, the “invention” of the obesity epidemic, or those with a cultural and anthropological basis are absent in the training of healthcare providers toward a clinical/assisting approach to obesity. Most studies stem from countries without a universal health system, evincing that obesity has meanings in these countries unlike those of a “Brazilian-style” PHE. Thus, the great need to encourage the dissemination of research in the area and of considering the use of PHE as urgent.

The strengths of this review include its comprehensive bibliographical research in various electronic databases via a rigorous methodology suggested by JBI^7^and Prisma-ScR^8^. Note also its novelty in view of the scarcity of publications in the national and international literature which focused on PHE focused on obesity care. Worldwide, the use of these syntheses is considered a priority to formulate well-informed and effective policies^24 , 44^.

### Limitation

This study shows limitations regarding the scarcity of evidence, especially for Latin America and mainly for Brazil, regions which have consolidated PHE. Moreover, this scope review was a huge undertaking and our results include research only up to October 2020.

## CONCLUSION

This scope review showed a scarcity of research in its area of interest and that most PHE initiatives for obesity care show a traditional teaching methodology centered on transmission of information techniques, punctual actions, and disciplinary fragmentation, unlike what PHE proposes. It also suggests that the concept of obesity is still mostly linked to a biomedical bias, although the literature has gradually included other causal approaches, and that short-term research fails to promote changes in the field of procedural content, making learning and organizational changes unfeasible. Future research should study continuous initiatives focused on professionals’ daily work, recognizing their problems as real and strategic knowledge triggers, considering the notion of network, line of care, integrality of care, and food and body cultures.

**Table 1:** Characteristics of the included studies (n = 10).

Authors (year)	Country	Type of national health system	Funding source type	Journal discipline	Article type	Study design	Context/setting	Population
Whitaker et al. (2004)^10^	USA	No free or universal healthcare	Public-sponsored	Science of Nutrition	Original article	Quasi-experimental study	Kentucky 44th Annual Maternal and Child Health Conference	Nutritionists, nurses, and social workers
Katz et al. (2005)^11^	Israel	Free and universal	Not reported	Training of healthcare providers	Original article	Mixed study	Primary care	Nutritionists, nurses, physicians, psychologists, physiotherapists, and physiologists
Stark et al. (2011)^12^	USA	No free or universal healthcare	Public-sponsored	Nutrition education	Original article	Quasi-experimental study	Primary care	Nutritionists
McPherson et al. (2012)^13^	USA	No free or universal healthcare	Not reported	Public health	Original article	Mixed study	Primary care	Nutritionists, nurses, and physicians
Sarayani et al. (2012)^14^	Iran	Free and universal	Not reported	Training of healthcare providers	Original article	Randomized clinical trial (RCT)	Community pharmacy	Pharmaceutical workers
Campbel-Scherer et al. (2014)^15^	Canada	Free and universal	Privately-sponsored	Scientific study of methods for health	Original article	Mixed study	Primary care	Nutritionists and physical educators
Mainor et al. (2014)^16^	USA	No free or universal healthcare	Public-sponsored	Public health	Original article	Quasi-experimental study	Primary care	Nutritionists, nurses, and physicians
Vasudevan et al. (2016)^17^	USA	No free or universal healthcare	Privately-sponsored	Health disparities based on race and ethnicity	Original article	Quasi-experimental study	Primary care	Physicians
Joshua (2017)^18^	USA	No free or universal healthcare	Non-sponsored	Not applicable (dissertation)	Original article	Quasi-experimental study	Primary care	Nurses and physicians
Sanchez-Ramirez et al. (2018)^19^	Canada	Free and universal	Non-sponsored	Training of healthcare professionals	Original article	Quasi-experimental study	Primary care	Nutritionists, nurses, physicians, and pharmaceutical, dental, and social workers
